# Plasma-Engineered CeO*_x_* Nanosheet Array with Nitrogen-Doping and Porous Architecture for Efficient Electrocatalysis

**DOI:** 10.3390/nano14020185

**Published:** 2024-01-13

**Authors:** Zhou Wang, Tong Li, Qi Wang

**Affiliations:** Key Laboratory of Liquid-Solid Structural Evolution and Processing of Materials of Ministry of Education, School of Materials Science and Engineering, Shandong University, Jinan 250061, China; wangzhou@sdu.edu.cn (Z.W.); litong@sicc.cc (T.L.)

**Keywords:** nitrogen-doping, oxygen vacancy, plasma, CeO_2_, electrocatalysis

## Abstract

Surface engineering has been proved efficient and universally applicable in improving the performance of CeO_2_ in various fields. However, previous approaches have typically required high-temperature calcination or tedious procedures, which makes discovery of a moderate and facile modification approach for CeO_2_ an attractive subject. In this paper, porous CeO_2_ nanosheets with effective nitrogen-doping were synthesized via a low-temperature NH_3_/Ar plasma treatment and exhibited boosted hydrogen evolution reaction performance with low overpotential (65 mV) and long-term stability. The mechanism of the elevated performance was investigated by introducing Ar-plasma-treated CeO_2_ with no nitrogen-doping as the control group, which revealed the dominant role of nitrogen-doping by providing abundant active sites and improving charge transfer characteristics. This work illuminates further investigations into the surface engineering methodologies boosted by plasma and the relative mechanism of the structure–activity relationship.

## 1. Introduction

In recent years, with the increasing attention paid to environmental pollution and sustainable development issues, hydrogen energy has received widespread attention as a sustainable and clean substitute for fossil fuels by virtue of its high energy density and pollution-free combustion products [1,2,3,4]. Among various approaches for hydrogen production, electrocatalytic water splitting through hydrogen evolution reaction (HER) is believed to be a promising strategy with great application prospects and competitiveness [5,6,7]. The active electrode material, which dominantly determines the performance of the electrocatalytic devices, involves numerous kinds of emerging materials [5,6,7,8]. Noble metals, represented by Pt, have been universally acknowledged as excellent HER catalysts. However, the disadvantages of high cost, low long-term reliability, and limited reserves hinder their practical applications [9].

CeO_2_, as a common transition metal oxides catalyst, has two distinctive characteristics: one is that CeO_2_ prepared by conventional methods usually has a certain amount of oxygen vacancies (OVs), and the other is the flexible and reversible conversion between Ce^3+^ and Ce^4+^ oxidation states [10]. These two characteristics are conducive to achieving better electrocatalytic performance, but the low concentration of naturally generated Ce^3+^ and OVs always restrict the performance improvement of CeO_2_-based electrocatalysts [11]. Considering the heterogeneous reactions that take place on the surface of active materials, surface engineering has been applied as a popular approach to boost the catalytic activity of CeO_2_ [10,12,13,14,15]. Among various surface engineering strategies, doping can effectively boost activity through the regulation of the electronic structure and the chemical bonding conditions. In regard to oxides, nitrogen-doping has attracted special interest and has been confirmed beneficial in three aspects as follows: (1) the incorporation of nitrogen facilitates electron transfer and drives down the Schottky barrier between catalyst and electrolyte, thereby lowering the HER overpotential [16]; (2) the adsorption/desorption energy of reaction intermediates can be regulated by the injected N element, hence improving the reaction kinetics [17]; (3) the lower electronegativity of doped N element can raise the percentage of covalent bonds, leading to the enhancement of environmental, chemical, and structural stability, hence strengthening corrosion resistance to acidic or alkali solutions [18]. In addition, morphology modification is another common strategy which affects active reaction area, ion transfer, and surface energy. Porous structures with adequate active reaction sites and facilitated ion transfer characteristics are typically thought to be advantageous for electrocatalysis [19,20]. Previous studies have demonstrated that the fabrication of porous structure and the introduction of nitrogen-doping can significantly improve the electrochemical performance of CeO_2_. However, the strategies reported always entail calcination at high temperatures [21,22], which is prone to cause agglomeration in the pristine nanostructure and produce detrimental impacts on the final performance.

In this study, NH_3_/Ar plasma treatment was implemented to conduct surface engineering on hydrothermally constructed CeO_2_ nanosheets. Profiting from the energetic and activated plasma, porous CeO_2_ nanosheets with effective nitrogen-doping were produced at a low temperature. To validate the synergistic effect of etching and nitrogen-doping, systematic characterizations, encompassing morphology, phase, electron structure, and HER performance, were conducted on CeO_2_ nanosheets before/after NH_3_/Ar or pure Ar plasma treatment. Additionally, the mechanism underlying the superior HER performance was explored. Our work not only introduces a facile method to develop high-activity materials, but also reveals the mechanism in a kinetic thermodynamic view, which provides a valuable basis for future research on surface engineering with excellent catalytic performance. 

## 2. Materials and Methods

**Scheme 1:**
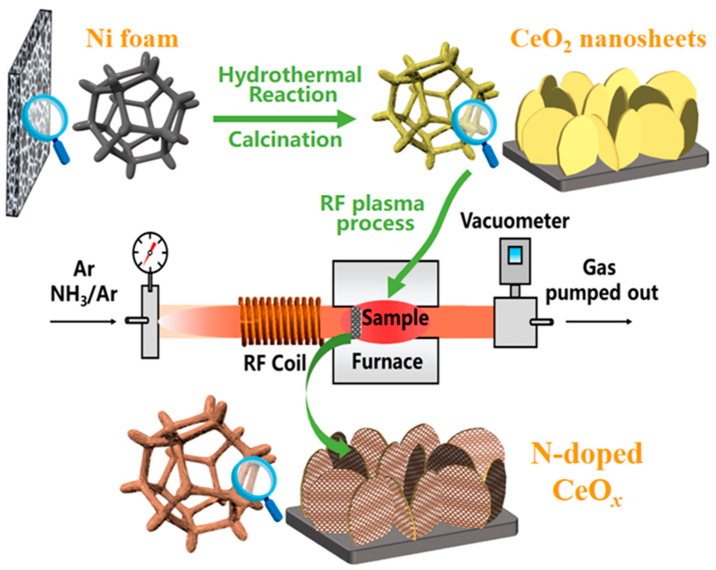
Schematic diagram of the fabrication procedures.

Scheme 1 depicts the fabrication procedures, which include the hydrothermal process and subsequent plasma treatment. 

### 2.1. Direct Growth of CeO_2_ Nanosheets on Ni Foam

Ni foam was cut into pieces with a fixed size of 2 cm × 3 cm which were ultrasonically cleaned for 15 min each with acetone, ethanol, and deionized water. Subsequently, the Ni foam substrates were soaked in 2M HCl for 10 min to etch the oxide layer and finally cleaned several times until the pH was neutral before storing in ethanol for further use.

CeO_2_ nanosheets were grown in situ on nickel foams via a hydrothermal method. In detail, 0.7 mmol Ce(NO_3_)_3_·6H_2_O, 3.5 mmol urea, and 2.8 mmol NH_4_F were dissolved in 35 mL distilled water under magnetic stirring in a 70 mL Teflon-lined stainless-steel autoclave. A piece of pre-cleaned Ni foam was transferred into the autoclave and immersed into the mixed solution. After being heated in an electric oven at 120 °C for 5 h and naturally cooled down to room temperature, the autoclave was unsealed to take out the Ni foam. In order to remove the residual solution and weakly adhered powder on the surface, the Ni foam was thoroughly washed with deionized water under ultrasonication and dried at 70 °C for 6 h. Afterwards, the samples were annealed in air at 500 °C for 2 h to transform the Ce-precursor into CeO_2_. The mass loading of CeO_2_ was determined to be approximately 8.3 mg/cm^2^ by comparing the mass difference before and after deposition. As a control experiment, a bare Ni foam received the same heat treatment. 

### 2.2. Plasma-Induced Fabrication of N-Doped CeO_x_

The N-doped CeO_2_ nanosheets were fabricated through treating samples in the NH_3_/Ar (NH_3_:Ar = 1:9) plasma induced by a 13.56 MHz radio frequency (RF) source. Firstly, the Ni foam assembled with CeO_2_ nanosheets was fixed vertically in the plasma reactor chamber, which was vacuumed until achieving a base pressure of 1 × 10^−5^ Pa. Then, the pressure of the chamber was adjusted by controlling the NH_3_/Ar gas flow and stabilized at 40 ± 1 Pa. The RF plasma process was operated at a working power of 250 W. The doping amount of N and the plasma etching effect could be controlled by regulating the heating temperature and RF time. The N-doped CeO_2_ nanosheets obtained via plasma-induced fabrication were denoted as N-CeO_2_.

As control experiments, bare nickel foam after calcination was denoted as Ni foam. Bare nickel foam which had undergone the same calcination and RF plasma treatment as N-CeO_2_ was denoted as N-Ni foam. The CeO_2_ nanosheets treated by high-quality pure Ar plasma with the same parameters as N-CeO_2_ was denoted as Ar-CeO_2_.

### 2.3. Characterization

Scanning electron microscopy (SEM) was performed on an ultra-high-resolution field-emission scanning electron microscope (JEOL 7800F, from Tokyo, Japan). JEM-2100F (Tokyo, Japan) operated at an accelerating voltage of 200 kV and was used to capture transmission electron microscopy (TEM) pictures. The crystallographic structure was studied by analyzing X-ray diffraction (XRD) patterns recorded by a Rigaku D/Max-KA diffractometer (Bruker from Billerica, Mass., USA), which was equipped with Cu Kα radiation (λ = 1.5418 Å). The chemical composition and valence state were characterized by X-ray photoelectron spectra (XPS) conducted on an Axis Supra (Kratos from Manchester, UK) using Mg Kα radiation of 1253.6 eV.

### 2.4. Electrochemical Measurements

An IVIUM Vertax (Eindhoven, Netherlands) electrochemical workstation was applied to perform the electrochemical measurements. The electrochemical performances were studied via a series of electrochemical testing approaches in a three-electrode system composed of a sample (working electrode), platinum (counter electrode), and the Hg/HgO (reference electrode) in 1.0 M KOH aqueous electrolyte at room temperature.

The performances of electrocatalytic hydrogen evolution were evaluated by the linear sweep voltammetry (LSV) method. The as-measured potential (*E_Hg/HgO_*), which used the Hg/HgO electrode as the reference, was transformed into the potential versus the reversible hydrogen electrode (RHE) using the following formula: *E_RHE_* = *E_Hg/HgO_* + 0.0591 × *pH* + 0.098. 

Electrochemical impedance spectroscopy (EIS) was recorded at an over potential of 100 mV (vs. RHE) in a frequency range of 100 kHz ~ 0.01 Hz with a constant amplitude of 5 mV. The electrochemical active surface areas (EASA) of different electrodes were evaluated by the double layer capacitance values (C_dl_), which were derived from fitting their CV curves in the potential range of −0.8 ~ 1.5 V at different scan rates (5 ~ 25 mV s^−1^). All tests were conducted without iR compensation.

## 3. Results and Discussion

### 3.1. Physicochemical Properties

According to previous reports, the effect of plasma treatment mainly depends on the RF time, heating temperature, plasma power, atmosphere, etc. [23,24,25]. In this work, in an effort to optimize the electrocatalytic performance of N-CeO_2_, the influence of RF time and heating temperature were chosen to be preferentially studied based on morphology characterization and systematic electrochemical testing (Overpotential, Tafel slope, Nyquist plot, Electrochemical active surface area). Finally, the optimal RF time and heating temperature were determined as 20 min and 300 °C, respectively (See Supporting Information Section S1 and S2 for detailed discussion). In the subsequent discussion, N-CeO_2_ refers in particular to CeO_2_ nanosheets plasma-treated at 300 °C for 20 min in NH_3_/Ar.

**Figure 1:**
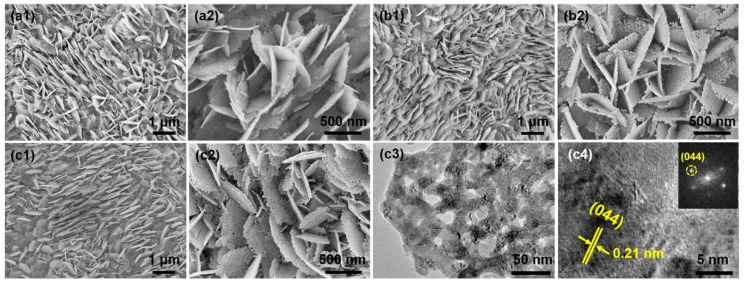
SEM images of (**a1**,**a2**) pristine CeO_2_, (**b1**,**b2**) Ar-CeO_2_, (**c1**,**c2**) N-CeO_2_. (**c3**,**c4**) HRTEM images of N-CeO_2_.

During the NH_3_/Ar plasma treatment, two mechanisms, including nitrogen-doping and etched porous structure, for improving catalytic activity were introduced simultaneously [19]. In order to distinguish the contribution of each mechanism in the HER performance, a CeO_2_ nanosheet array treat in pure Ar gas (Ar-CeO_2_) at the same temperature and time was fabricated and set as the control group, since an identical etching effect accompanied with no nitrogen-doping was involved. In order to figure out the influence of plasma on the morphology, field-emission scanning electron microscopy (FESEM) images of pristine CeO_2_, Ar-CeO_2_, and N-CeO_2_ were recorded (Figure 1). For pristine CeO_2_, homogeneous nanosheets with a diameter of ~1 μm and rough edge were vertically assembled on an Ni framework. After plasma treatment in pure Ar, the nanosheets basically maintained the origin morphology at low magnification. But detailed scope shows a porous and cracked structure under the bombardment of plasma-induced energetic particles, especially for the edge area with barely linked nanoparticles. N-CeO_2_ displays an identical porous structure and broken edge to Ar-CeO_2_, revealing a comparable etching effect. The TEM image of N-CeO_2_ (Figure 1c3) also displays porous structure with a pore diameter of 10–30 nm. The high-resolution TEM (HRTEM) image (Figure 1c4) exhibits an interplanar distance of 0.21 nm, which can be ascribed to the (044) facet of CeO_2_. The calibration of the selected-area electron diffraction (SAED) pattern (inset of Figure 1c4) further confirms the composition.

**Figure 2:**
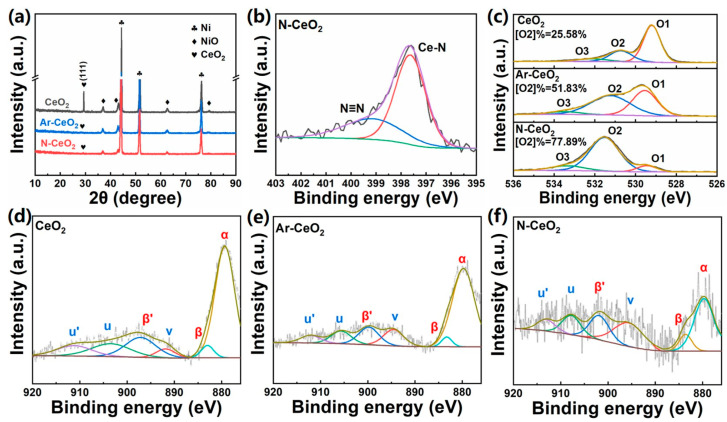
(**a**) XRD patterns of CeO_2_, Ar-CeO_2_, and N-CeO_2_. (**b**) High-resolution N 1s XPS spectra of N-CeO_2_. (**c**) High-resolution O 1s XPS spectra of CeO_2_, Ar-CeO_2_, and N-CeO_2_. High-resolution Ce 3d XPS spectra of (**d**) CeO_2_, (**e**) Ar-CeO_2_, and (**f**) N-CeO_2_.

In order to discover the effect of plasma treatment on the crystal phase, X-ray diffraction (XRD) characterizations were conducted on CeO_2_, Ar-CeO_2_, and N-CeO_2_ for comparison. As illustrated in Figure 2a, all samples show identical patterns with major peaks fitting well with Ni and NiO, which originate from the Ni foam substrate. The abnormal signal of NiO was caused by the oxidation of Ni during the calcination in air. In addition to the standard pattern of Ni and NiO, one peak located at 29.3° can be detected in the XRD pattern of CeO_2_, which is in good accordance with the characteristic signal from (111) crystal planes in the fluorite structure (face-centered cubic, FCC) of CeO_2_ crystal with an Fm-3m space group (PDF #34-0394). The strong intensity and narrow width of the peak indicates the considerable loading mass and good crystallinity of CeO_2_. However, the characteristic peak cannot be detected in the XRD patterns of Ar-CeO_2_ and N-CeO_2_. Since the SEM images in Figure 1 have confirmed the identical distribution of CeO_2_ nanosheets, the absence of an XRD peak can be ascribed to the low crystallinity caused by the plasma bombardment, which disrupts the long-range order of the lattice. Detailed investigation was carried out based on the magnified patterns within a diffraction angle of 24°–34°, as shown in Figure S5. After the Ar plasma etching, the original (111) peak collapses into a weak bump, which can barely be identified from the background. The central position of the signal seems to show a slight shift towards lower angle direction, implying an expansion of the lattice after plasma treatment, which can be attributed to the repulsive power of charged OVs. Compared to Ar-CeO_2_, the more recognizable bump of N-CeO_2_ can possibly be attributed to the relatively lower ionization capability of NH_3_, which weakened the etching effect of plasma. In addition, a reverse shift in the bump towards a high-angle direction was observed and can be explained by the injected nitrogen atoms, which are able to consume the electrons previously located at the OVs and, thus, to some extent eliminate the repulsive effect on the lattice and recover the pristine lattice constant.

Since the electron transportation from OVs or doped nitrogen atoms to adjacent Ce atoms directly changes the electronic structure, X-ray photoelectron spectroscopy (XPS) was applied as an effective approach in our investigation. The wide XPS survey spectra of CeO_2_, Ar-CeO_2_, and N-CeO_2_ (Figure S6) confirm the identical element composition of Ce and O for all samples, except for the detection of N in the spectrum of N-CeO_2_ with a considerable concentration of 11.7%. The introduced N element was further analyzed by the high-resolution N 1s spectrum, which can be deconvoluted into two characteristic peaks (Figure 2b). The sharp peak centered at 397.6 eV corresponds to the chemical bonding between Ce and incorporated N (Ce-N), while the broad one originates from the absorbed N_2_ (N≡N) [26,27]. The Ce-N confirms that N elements were successfully introduced into the CeO_2_ lattice. 

Previous reports have proved that plasma-induced etching and doping are prone to create OVs on the surface of metal oxides, which usually plays an important role in achieving superior performance [28,29,30]. Therefore, the high-resolution O 1s and Ce 3d spectra of CeO_2_, Ar-CeO_2_, and N-CeO_2_ were investigated (Figure 2c–f). As depicted in Figure 2c, the O 1s XPS spectra of all samples display three deconvoluted peaks at O1, O2, and O3 with binding energy values of ~529.2, ~530.7, and ~532.1 eV. The O1 peak represents the Ce–O bond and the O2 peak is assigned to the OVs, whereas the surface-adsorbed hydroxyl groups are responsible for the O3 peak [31]. Therefore, the concentration of OVs can be evaluated by the ratio of the peak area of O2 in the total peak area of (O1 + O2 + O3). According to the fitted spectra in Figure 2c, the proportion of O2 in Ar-CeO_2_ (51.83%) and N-CeO_2_ (77.89%) is obviously higher than that in CeO_2_ (25.58%), suggesting that extra OVs are generated during the plasma treatment. The highest OV concentration in N-CeO_2_ can be explained as follows. When lattice O atoms escape from the lattice, they leave previously bound electrons in the oxide and form charged OVs, which create localized electron fields and set extra constraint on the neighboring O atoms. Therefore, the detachment of O atoms becomes harder with the increasing concentration of OVs. When it comes to N-CeO_2_, the released electrons can be consumed by the injected N atoms, thus weakening the electron fields and producing more OVs.

The formation of OVs in metal oxides is usually accompanied by the generation of metal ions with lower valence states [32], so the Ce 3d spectra CeO_2_, Ar-CeO_2_, and N-CeO_2_ were also recorded for comparison. As shown in Figure 2d–f, all Ce 3d spectra can be deconvoluted into two sets of overlapped peaks. The peaks corresponding to Ce^3+^ are labeled with α, β, and β′, while peaks related to Ce^4+^ are denoted as v, u, and u′ [33]. The concentration of Ce^3+^ can be estimated by the peak area ratio of Ce^3+^-related peaks to all fitted peaks, which was calculated to be 65.8%, 78.3%, and 62.1% for CeO_2_, Ar-CeO_2_, and N-CeO_2_, respectively. The increased concentration of Ce^3+^ in Ar-CeO_2_ is in accord with the previously discussed OVs. However, the amount of Ce^3+^ in N-CeO_2_ sharply decreased, which is inconsistent with the highest content of OVs. The low concentration can be explained by the bonding between N and Ce, which promotes the transition from Ce^3+^ to Ce^4+^.

### 3.2. HER Performance

The electrocatalytic capability of CeO_2_, Ar-CeO_2_, and N-CeO_2_ toward HER was evaluated by three key indexes (overpotential, Tafel slope, and long-term durability), whereas Ni foam and N-Ni foam were also involved to avoid interference from the substrate. 

**Figure 3:**
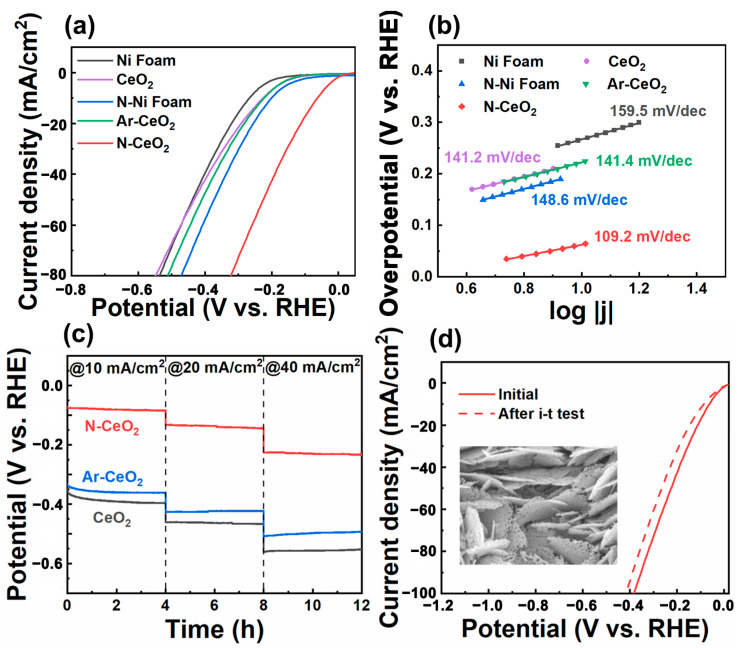
HER performance: (**a**) LSV curves, (**b**) Tafel plots, (**c**) long-term chronopotentiometric curves at different current densities, and (**d**) LSV curves of N-CeO_2_ before and after chronopotentiometric test. Inset is the SEM image of N-CeO_2_ after chronopotentiometric test. The current densities in all figures are based on geometric area.

Firstly, overpotential, which represents the actual voltage required to reach a certain current density that exceeds the theoretical voltage, was investigated by a linear sweep voltammetry (LSV) test. As presented in Figure 3a, bare Ni foam displays unsatisfactory performance with a large overpotential of 370 mV at the current density of 10 mA/cm^2^, while the hydrothermal growth of CeO_2_ nanosheets improves the performance by reducing the overpotential to 230 mV, which indicates that CeO_2_ nanosheets have considerable catalytic activity towards HER. Contrary to our expectations, Ar-CeO_2_ with abundant OVs and Ce^3+^ exhibits an almost overlapping LSV curve over that of CeO_2_ at low potential range and shows the same value of overpotential, indicating that OVs and Ce^3+^ are not able to perform well as active HER sites in Ar-CeO_2_. Favorably, N-CeO_2_ achieves the lowest overpotential of 65 mV, indicating its superior HER activity. Meanwhile, N-Ni foam displays a lower overpotential (~200 mV) than untreated Ni foam. Although both N-Ni foam and N-CeO_2_ receive decreased overpotential, the effect of NH_3_/Ar-plasma treatment is much more obvious on CeO_2_. Considering the fact that most of substrate is covered by the assembled CeO_2_ nanosheets, it is reasonable to draw the conclusion that the superior overpotential of N-CeO_2_ mainly originates from nitrogen-doping sites, which are capable of catalyzing HER.

Tafel slope is a crucial metric in evaluating the kinetic characteristics of reactions and the activity of electrochemical catalysts. Normally, catalysts with a lower Tafel slope are believed to have higher activity and better ability to accelerate the reaction rate. Based on the linear part in the low potential range of the LSV plots (Figure 3a), the Tafel slope of each sample can be acquired through linear fitting in the coordinate system of log|j|–overpotential (Figure 3b). In a general view, electrodes with CeO_2_ loading show a lower Tafel slope than bare Ni foam, whether before or after plasma treatment, demonstrating the superior HER dynamics of CeO_2_. The values of Tafel slopes are in the range of 110–150 mV/dec for all CeO_2_-involved samples, indicating the Volmer–Heyrovsky mechanism. Among them, N-CeO_2_ achieves the lowest Tafel slope of 109.2 mV/dec, which is much better than the similar values of CeO_2_ (142.7 mV/dec) and Ar-CeO_2_ (141.4 mV/dec), confirming its superior HER dynamics.

Long-term durability, as a crucial factor to evaluate the practicality of electrocatalysts, was investigated through chronopotentiometric testing at different current densities. As can be seen in Figure 3c, all CeO_2_-involved samples were tested at the current densities of 10, 20, and 40 mA/cm^2^, with each current density for 4 h. During the 12 h testing time, the voltage of all samples remained almost steady at each current density level. Significantly, the overpotential of N-CeO_2_ remained an apparent advantage at each current level during the whole test. In order to verify the influence of long-term testing, the LSV plot of N-CeO_2_ was recorded after the test and compared with the initial plot in Figure 3d. The LSV plot after the test shows a slight shift towards a negative potential direction and gains an increased overpotential of ~90 mV, which is acceptable as an experimental electrode. The morphology of N-CeO_2_ remains a porous nanosheet array, indicating that the larger overpotential is not due to structure collapse. 

At last, the performance of our N-CeO_2_ was compared with previously reported HER catalysts, most of which are CeO_2_-based materials [34,35,36,37,38,39,40,41,42]. As listed in Table 1, the overpotential and Tafel slope of N-CeO_2_ are superior or comparable to most of the previously developed CeO_2_-based catalysts, even when they are incorporated with other functional substance which may improve their conductivity or directly act as active components. Notably, Co_4_N-CeO_2_ and CoP-CeO_2_ exhibit extremely low overpotentials and Tafel slopes, which can be ascribed to the difference in their main active species and HER mechanisms. This mechanism can be judged from the low Tafel values of 61 mV/dec and 45 mV/dec, corresponding to the Heyrovsky or Tafel-rate-determining mechanism, respectively [39,40]. If these active substances were compounded with our N-CeO_2_ instead of normal CeO_2_, the HER performance would probably improve to the next level, considering the boosted performance of N-CeO_2_.

### 3.3. Mechanism Study 

Previous reports indicate that N-CeO_2_ is primarily affected by NH_3_/Ar plasma treatment in three ways: (1) creating porous structure and expanding active surface area; (2) introducing oxygen vacancies; (3) injecting nitrogen-dopant into CeO_2_ lattice. The first two effects also work on Ar-CeO_2_. In Section 3.2, the key role of N-doping and its leading contribution to the HER performance of N-CeO_2_ have been confirmed by comparative investigation, but its working mechanism is still unknown. To solve this problem, electrochemical active surface areas (EASA) and Nyquist tests were conducted.

**Figure 4:**
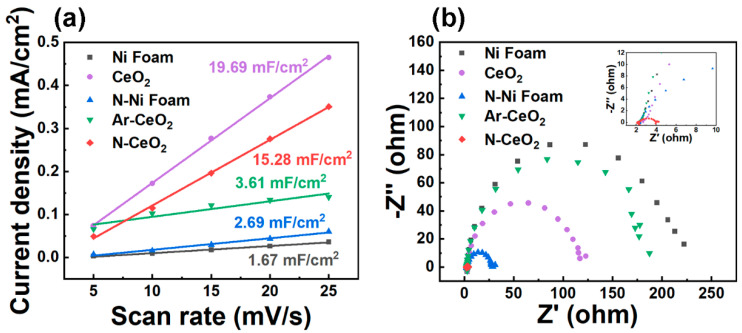
(**a**) The linear fitting plots of anode currents (derived from Figure S7) vs. scan rates for EASA evaluation. (**b**) Nyquist curves.

Firstly, the EASA tests were performed to evaluate the effect of etching and nitrogen -doping on the activation of reaction sites. The CV curves of various samples were recorded in the non-faradic regions (1.02–1.12 V) at various scan rates (5–25 mV/s), as illustrated in Figure S7. By linear fitting the anode current densities on each group of CV curves, the slopes of all sample were obtained in the coordinate system of scan rate vs. current density (Figure 4a). The EASA of each electrode is represented by the obtained slope in the form of double-layer capacitance since the double-layer capacitance is in direct ratio. Among all samples, the low EASA values of Ni foam and N-Ni foam are consistent with expectations, However, the sharply decreased EASA from 19.69 mF/cm^2^ (for CeO_2_) to 3.61 mF/cm^2^ (for Ar-CeO_2_) is unexpected, since the generation of a porous structure should create abundant extra sites. The “collapsed” EASA could be explained as follows. The etching effect of plasma indeed creates more exposed sites, but these sites are incapable of catalyzing the HER reaction, making them “inactive” sites. In that case, the disappointing HER performance of Ar-CeO_2_ is understandable. In regard to N-CeO_2_, although a similar etching effect of plasma passivates the surface sites, the nitrogen injection effectively activates some of the reaction sites and endows them with higher activity. Therefore, a superior HER performance can be achieved by N-CeO_2_ with a relatively lower EASA (15.28 mF/cm^2^).

Secondly, charge transfer behavior is also important for electrocatalysis, especially for semiconductors, such as N-CeO_2_. Thus, EIS characterization was conducted in a three-electrode system to study electron transportation behavior. As displayed in Figure 4b, the high-frequency part of each Nyquist plot differs significantly from each other in the diameter of the semicircle, which represents different charge transfer resistances (R_ct_). The largest R_ct_ value for Ni foam can be attributed to the surface oxidation during calcination at 500 °C. The R_ct_ of CeO_2_, Ar-CeO_2_, and N-CeO_2_ were determined to be 121.3 Ω, 193.5 Ω, and 2.1 Ω, respectively. The increased R_ct_ of Ar-CeO_2_ can be ascribed to the hindered charge transfer between electrode and electrolyte. As can be seen from the extremely low R_ct_ value of N-CeO_2_, nitrogen-doping effectively facilitates charge transfer, which also promotes the improvement in HER performance.

Above all, nitrogen incorporation, which activates surface reaction sites and facilitates charge transfer, plays the dominant role in achieving superior HER performance.

## 4. Conclusions

In summary, this work successfully developed nitrogen-doped CeO_2_ nanosheet arrays through low-temperature NH_3_/Ar plasma treatment and achieved an obviously improved HER performance. The systematic design of control groups and characterizations on reaction kinetics confirm the critical role of introducing nitrogen-doping, which promotes electron transfer and generates abundant electroactive sites. As a consequence, the optimized N-CeO_2_ electrode possessed a decreased overpotential of 65 mV vs. RHE at 10 mA/cm^2^ as well as a smaller Tafel slope of 109.2 mV/dec. Meanwhile, the N-CeO_2_ electrode also performed well in the long-term durability test. Given these properties, the as-constructed N-CeO_2_ electrode is superior to most of the reported CeO_2_-based HER catalysts. We believe this work not only introduces a favorable pathway for the modification of high-performance catalysts, but also offers a deeper understanding of the working mechanism of multiple influencing factors.

## Figures and Tables

**Table 1:** Comparison of N-CeO_2_ nanosheets with other HER catalysts in terms of overpotential at 10 mA/cm^2^ and Tafel slope.

Active Materials	Overpotential (mV vs. RHE)	Tafel Slope (mV/dec)	Ref.
N-CeO_2_	65	109.2	Our work
CeO_2_/Co(OH)_2_	317	144	[34]
3D CeO_2_/rGO	341	112. 8	[35]
Ni-CeO_2_/rGO	111	107.3	[36]
Ni_3_N-CeO_2_	80	122	[37]
Ni-S/CeO_2_	180	158	[38]
Co_4_N/CeO_2_	24	61	[39]
CoP/CeO_2_	43	45	[40]
NiFe nanotube	181	147	[41]
CoNi@N-C/rGO	510	133.7	[42]

## Data Availability

The data presented in this study are available on request from the corresponding author.

## Supplementary Materials

The following supporting information can be downloaded at: https://www.mdpi.com/article/10.3390/nano14020185/s1, Figure S1: SEM images of CeO_2_ nanosheets plasma-treated at 300 °C for different duration; Figure S2: (a) LSV curves, (b) Tafel plots, (c) EASA test and (d) Nyquist plots for CeO_2_ nanosheets plasma-treated at 300 °C for different duration; Figure S3: SEM images of CeO_2_ nanosheets plasma-treated for 20 min at different heating temperature; Figure S4: (a) LSV curves, (b) Tafel plots, (c) EASA test and (d) Nyquist plots for CeO_2_ nanosheets plasma-treated for 20 min at different heating temperature; Figure S5: Magnified XRD patterns within the diffraction angle of 24°–34°; Figure S6: Wide XPS survey spectra of CeO_2_, Ar-CeO_2_, and N-CeO_2_; Figure S7: CV curves under different scan rates (5–25 mV/s).
