# Supplementary material for: Plasma-Engineered CeOx Nanosheet Array with Nitrogen-Doping and Porous Architecture for Efficient Electrocatalysis
Source: Nanomaterials (Basel). 2024 Jan 13;14(2):185. doi: 10.3390/nano14020185 (PMC10821299; doi:10.3390/nano14020185)
Supplement: Supplementary file 1 [file nanomaterials-14-00185-s001.zip › nanomaterials-2797938-supplementary.pdf]

Supplementary Materials

Plasma-engineered CeO_x nanosheet array with nitrogen-doping and porous architecture for efficient electrocatalysis

Zhou Wang, Tong Li and Qi Wang *

Key Laboratory of Liquid-Solid Structural Evolution and Processing of Materials of Ministry of Education, School of Materials Science and Engineering, Shandong University, Jinan 250061, China; wangzhou@sdu.edu.cn (Z. W.); litong@sicc.cc (T.L.)
*Correspondence: wangqi1016@sdu.edu.cn

Section S1. Optimization on plasma treating time

As shown in Figure S1, CeO_2 electrodes treated in NH_3/Ar plasma for 20–40 min still maintained the uniform distribution of nanosheets with identical size to pristine CeO_2. The porous structure produced by the etching effect of plasma becomes more obvious with the increasing treating time. With a short treating time of 20 min, most pores gathered at the edge of nanosheets, because the relatively low crystallinity at the edge weakens the ability to resist etching. When extending the treating time to 30 min, a lot of pores emerges on the plane of nanosheets. However, a longer treating time of 40 min partially breaks the vertically grown nanosheets and some cracks can be found in the scope.

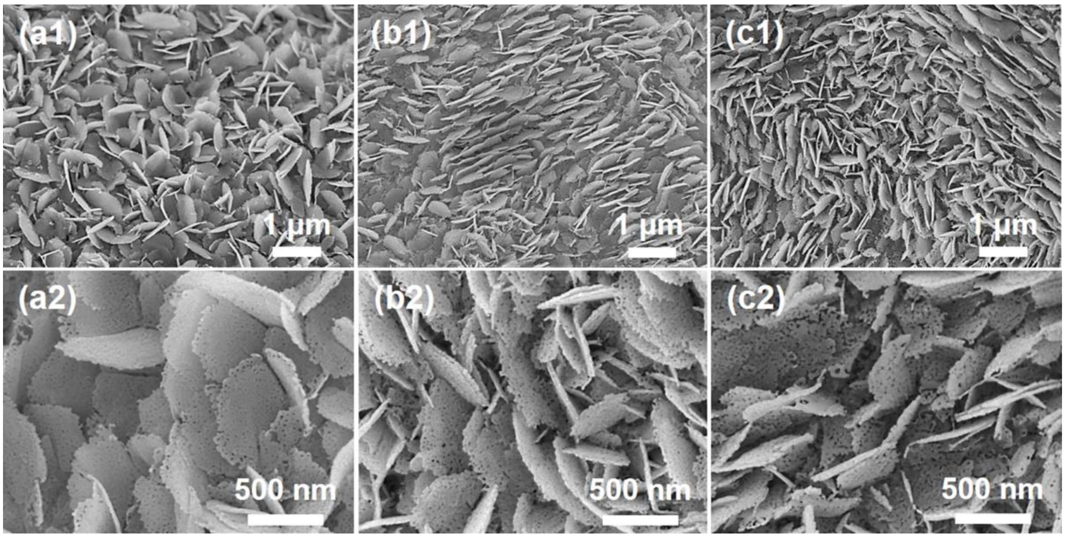

Figure S1. SEM images of CeO_2 nanosheets plasma-treated at 300 °C for (a1, a2) 10 min, (b1, b2) 20 min, (c1, c2) 30 min.

The HER performance of all samples is evaluated by the overpotential and Tafel slope. As shown in Figure S1a, with the increased treating time, the overpotential of N-CeO_2 increases first and then decreases. N-CeO_2-20min obtains the lowest overpotential of 65 mV, which is obviously lower than N-CeO_2-10min (160 mV) and N-CeO_2-10min (110 mV). The linear fitted Tafel plots in Figure S1b also displays that the minimum Tafel slope of 109.2 mV/dec is achieve by N-CeO_2-20min. In order to verify the origin of the optimum performance of N-CeO_2-20min, the electrochemical active surface areas (EASA) and Nyquist plots of all samples were investigated. As shown in Figure S1(c, d), N-CeO_2-20min obtains the largest EASA and smallest charge-transfer resistance, indicating its abundant active reaction sites and facilitated electron

transfer between electrode and electrolyte. Therefore, the plasma treating time was firstly determined to be 20 min.

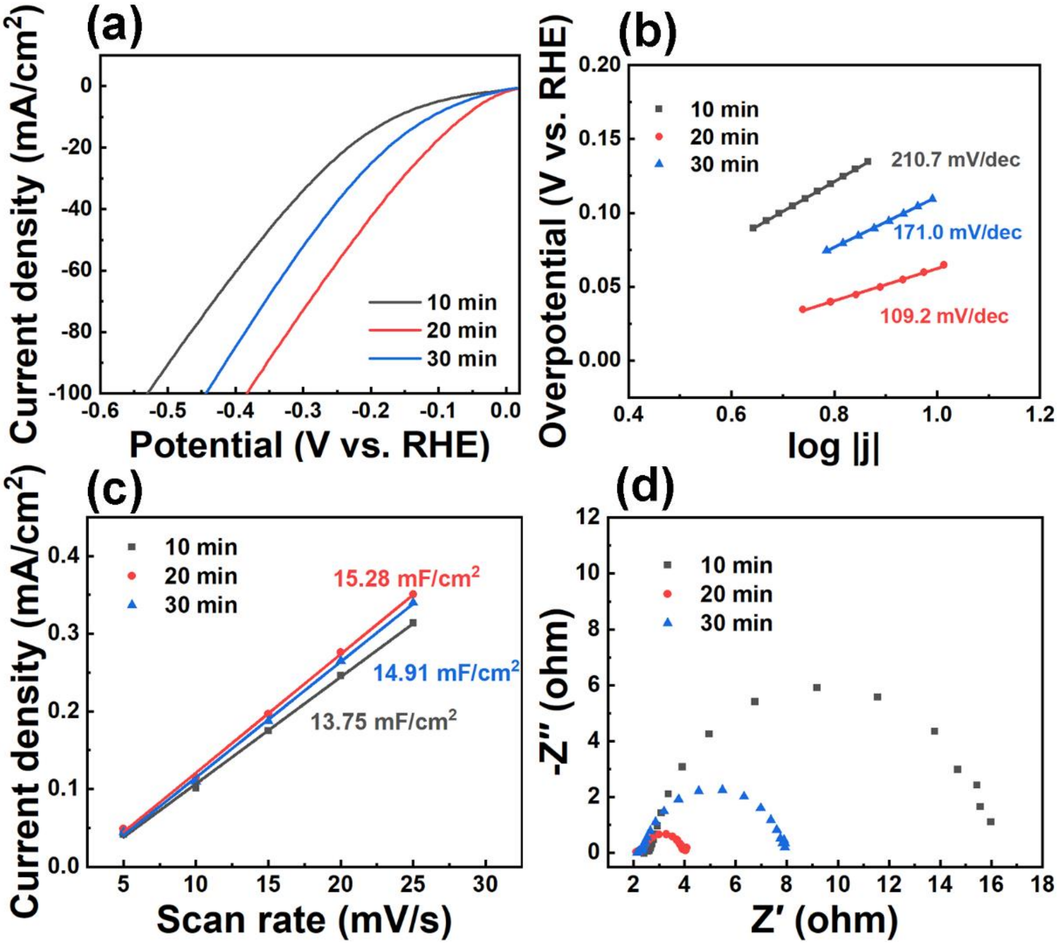

Figure S2. (a) LSV curves, (b) Tafel plots, (c) EASA test and (d) Nyquist plots for CeO_2 nanosheets plasma-treated at 300 °C for different duration.

Section S2. Optimization on heating temperature

As shown in Figure S3, the morphology of CeO_2 remains with no obvious pores after plasma-treated at 200 °C, which can be attributed to the poor atomic mobility at low temperature. The increased temperature endows atoms with high mobility, promoting the etching effect and the porous structure was produced at 300 °C and 400 °C. However, the plasma treatment at high temperature of 400 °C causes severe damage on pristine structure, making the nanosheets fragile and broken.

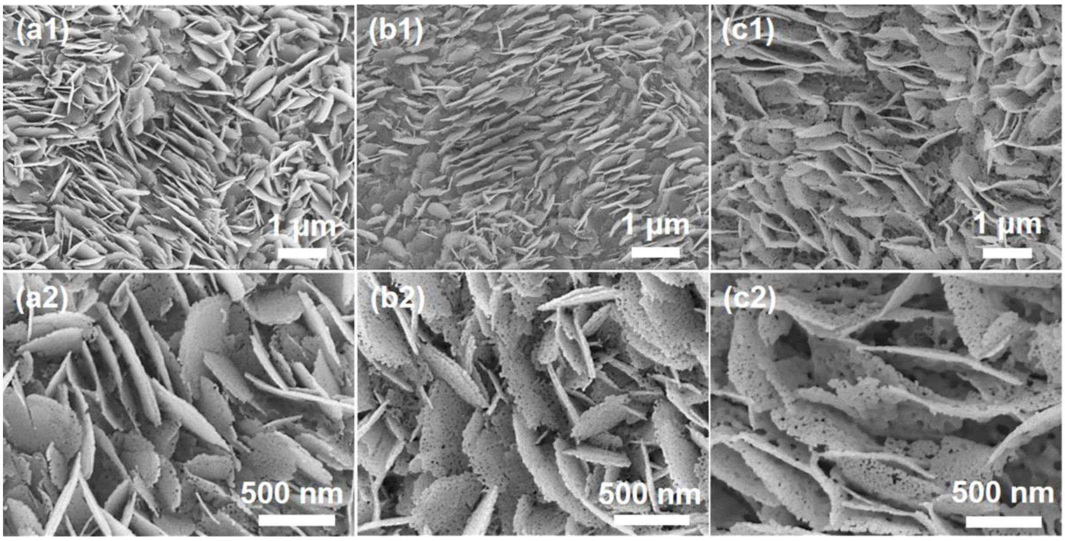

Figure S3. SEM images of CeO_2 nanosheets plasma-treated for 20 min at (a1, a2) 200 °C, (b1, b2) 300 °C, (c1, c2) 400 °C.

The influence of heating temperature on HER performance is identical with the treating time, as can be concluded by Figure 4. Low heating temperature leads to insufficient activation of reaction sites and high R_{ct}, while over high temperature induces the structure collapse and increases the R_{ct}. An appropriate heating temperature of 300 °C receives the best HER performance. Therefore, the optimum heating temperature was determined to be 300 °C.

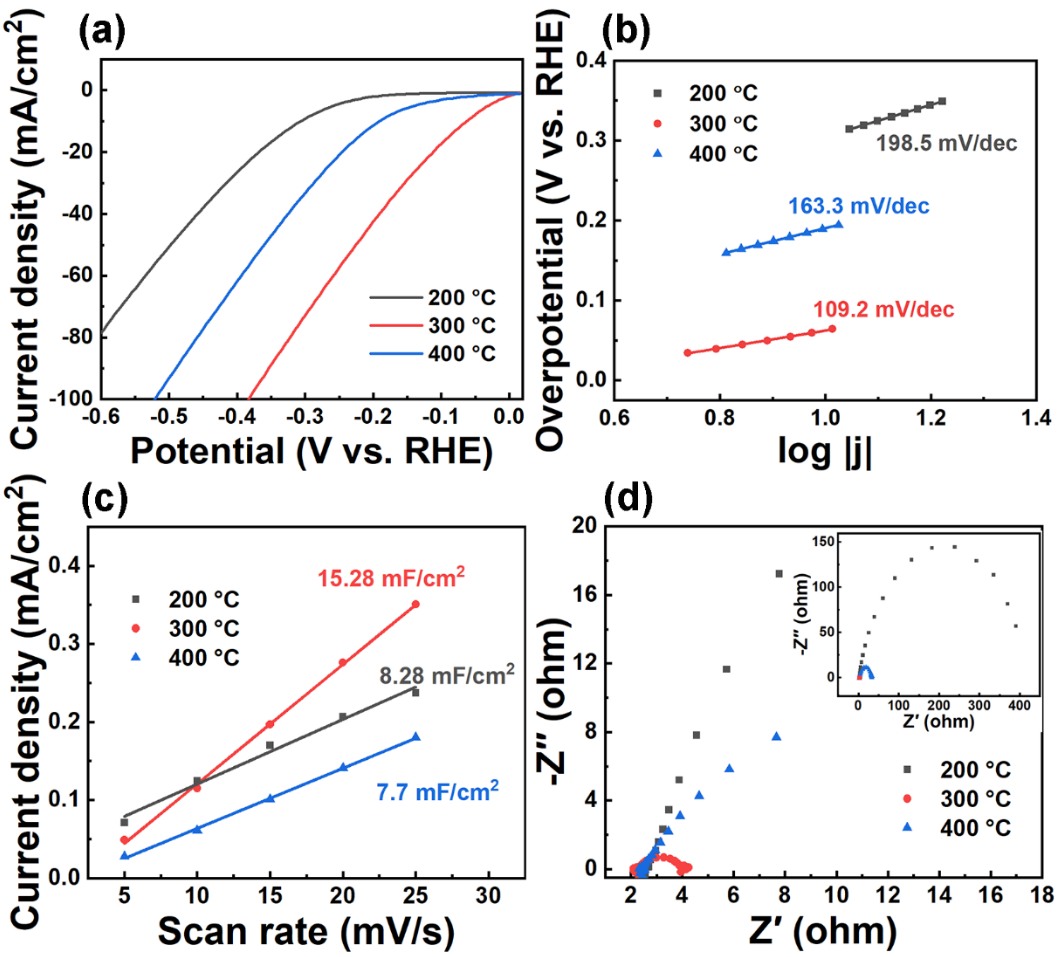

Figure S4. (a) LSV curves, (b) Tafel plots, (c) EASA test and (d) Nyquist plots for CeO_2 nanosheets plasma-treated for 20 min at different heating temperature.

Section S3. Other supplementary figures

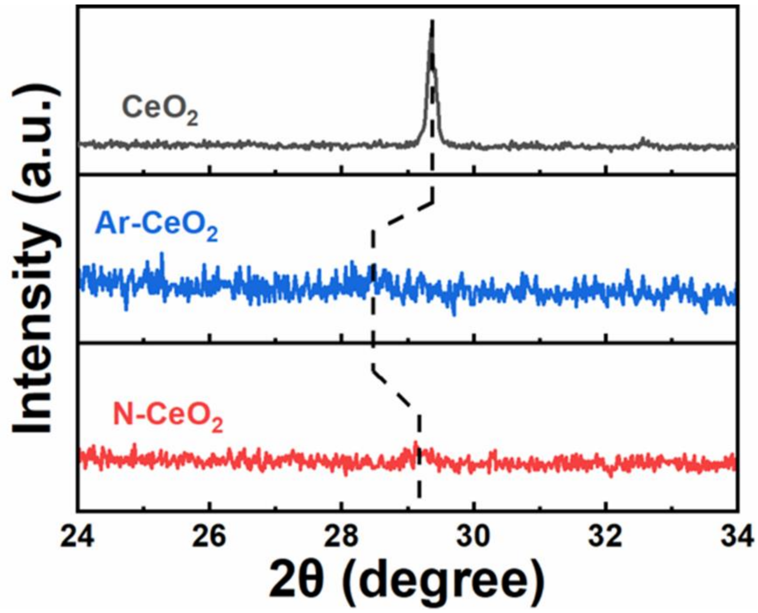

Figure S5. Magnified XRD patterns within the diffraction angle of 24°–34°.

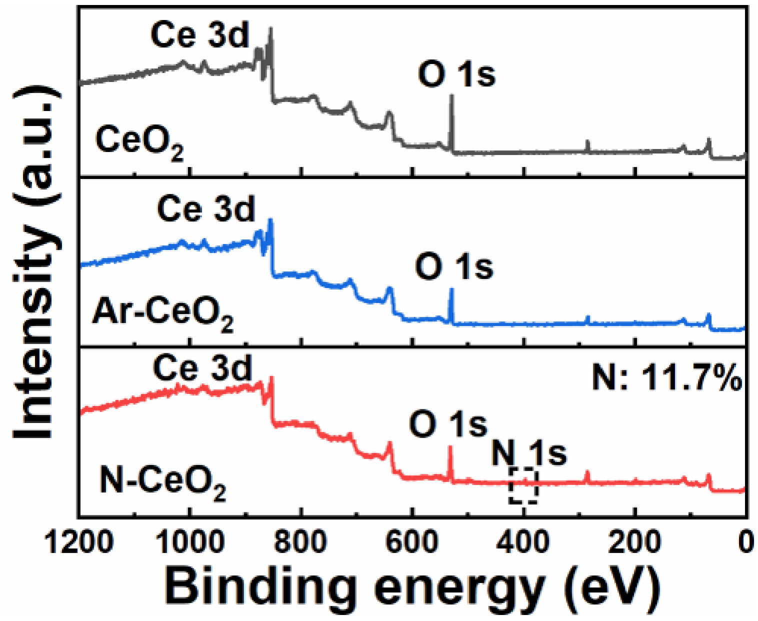

Figure S6. Wide XPS survey spectra of CeO_2, Ar-CeO_2 and N-CeO_2.

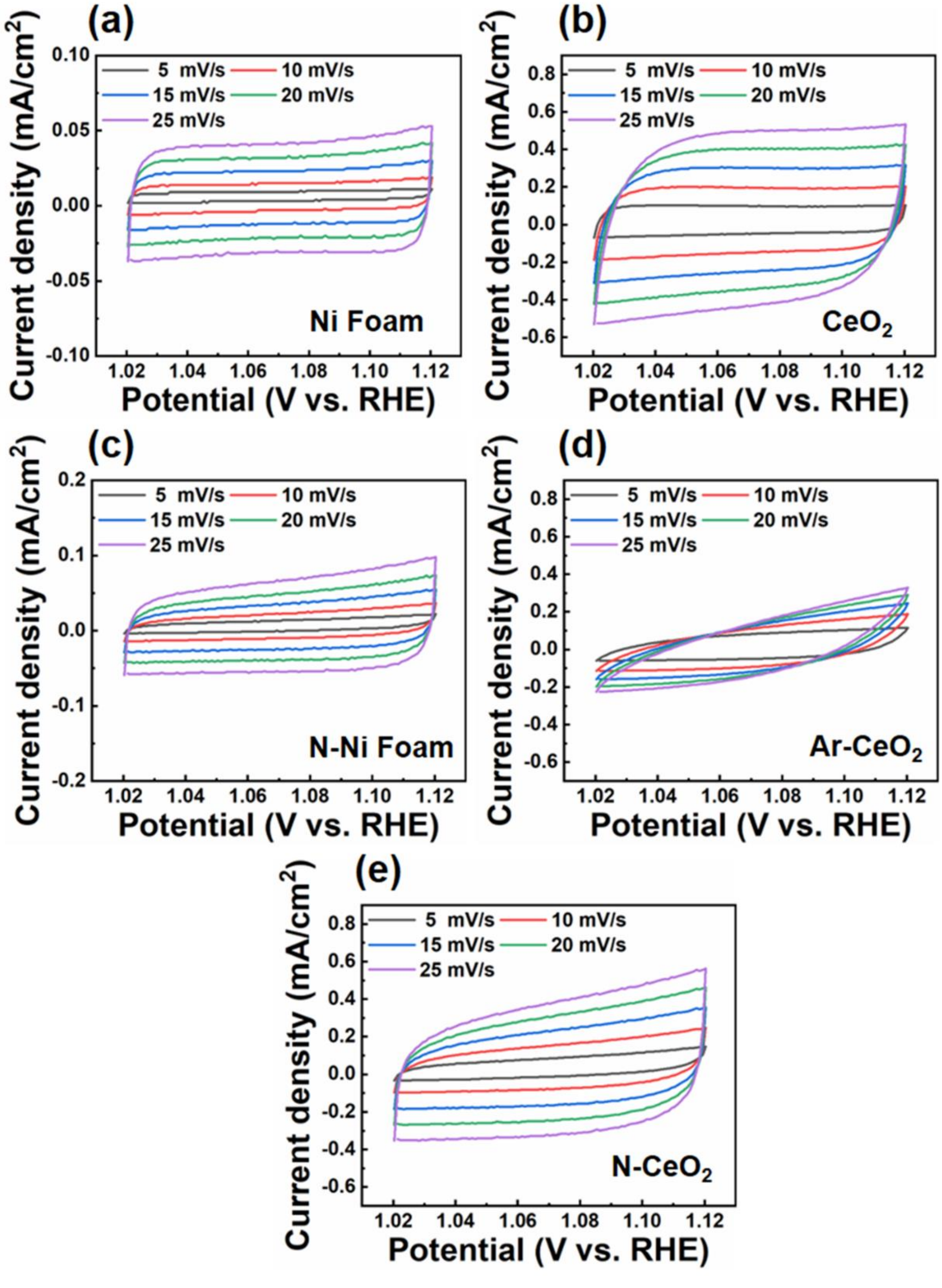

Figure S7. CV curves under different scan rates (5–25 mV/s).
